# Global visibility for global health: Is it time for a new descriptor in Medical Subject Heading (MeSH) of MEDLINE/PubMed?

**DOI:** 10.7189/03.010201

**Published:** 2013-06

**Authors:** Ana Marušić

**Affiliations:** Croatian Centre for Global Health and Department of Research in Biomedicine and Health, University of Split School of Medicine, Split, Croatia

A PubMed search using the term “global health” [All Fields] retrieves 8975 articles published up to the end of 2012. This is a large body of evidence for a new discipline in health research, defined in 2009 as an “area of study, research, and practice that places a priority on improving health and achieving equity in health for all people worldwide. Global health emphasizes transnational health issues, determinants, and solutions; involves many disciplines within and beyond the health sciences and promotes interdisciplinary collaboration; and is a synthesis of population–based prevention with individual–level clinical care” [1].

## PUBMED HISTORY OF GLOBAL HEALTH

**Figure 1:**
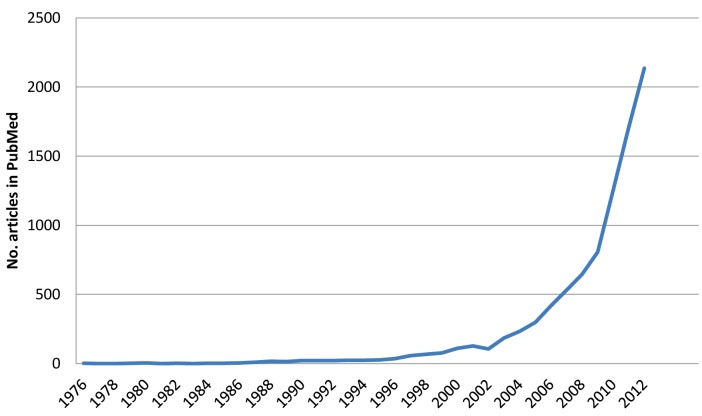
Number of articles indexed in PubMed which contained “global health” as a term anywhere in the text or bibliographical information.

According to PubMed, the first article that mentions global health was published already in 1966, describing “global health factors of importance” to military forces with worldwide engagement [2]. The term “global health” was again used 10 years later, in 1976, in an article that recognized blindness and its prevention as a global health problem; the article was published in a Swedish general medical journal [3]. From 1979, there is a steady flow of articles related to global health, with a major increase from 2006 onwards (**Figure 1**), paralleled by a dramatic increase in academic involvement in global health training programmes [4]. In 2010 and 2011, the number of articles containing the term “global health” went over a thousand, and then almost doubled in 2012, the year which closed with 2136 articles (**Figure 1**). At the time of finishing this viewpoint (search performed on 29 May 2013), the number of articles in less than the first 5 months of 2013 was 1165, indicating a continuing rapid growth of global health research.

## GLOBAL HEALTH AS A BIBLIOGRAPHICAL CONCEPT

Despite such volume of research output in global health, the term “global health” is not included in the Medical Subject Headings (MeSH) of the NLM – its controlled vocabulary thesaurus which NLM uses to index articles in MEDLINE [5]. NLM states that its indexers include new terms in MeSH “as they appear in the scientific literature or in emerging areas of research”. Search of MeSH for the truncated term “global” returns 52 terms, mostly related to protein biochemistry. Only 7 terms have some relevance to global health, such as “Global Warming” (introduced in 2010), “Disease Eradication” (introduced in 2012), “Carbon Footprint” (introduced in 2011), “World War I” and “World War II” (introduced in 2005), “Greenhouse Effect” (introduced in 1994) and “Transcultural Nursing” (introduced in 1992). It is interesting that “Global Warming” is a MeSH term although the number of articles using this term is almost 3 times smaller than that for “global health”. PubMed search for “global warming” (“global warming”[All Fields]) retrieves 3054 articles until the end of 2012, with the highest annual output of 504 articles in 2012.

## JOURNALS SPECIALIZED IN GLOBAL HEALTH

In addition to the lack of conceptualization of global health in the largest collection of medical literature, there are only 4 journals currently indexed in MEDLINE that have global health in their focus, according to the search of the Catalog of the National Library of Medicine (NLM) using the search strategy: “global health”[All Fields] AND (ncbijournals[All Fields] AND currentlyindexed[All]). These journals are *Pathogens and Global Health*, *Global Health Promotion*, *Global Health Action*, and *Globalization and Health*. The search of the NLM Catalogue for the journals indexed in PubMed Central and thus also available in PubMed [6] identifies 2 more journals: *Emerging Microbes & Infections* and *Journal of Global Health*.

## *JOURNAL OF GLOBAL HEALTH (JoGH)* – PRESENT AND FUTURE

Yes, one of 6 journals in PubMed dedicated to global health is *Journal of Global Health* (*JoGH*), the journal we started 2 years ago for reason described above – the need to communicate rapidly accumulating research on health issues with global impact [7]. Two years later, the data on journal visibility fully justify its launch. Particularly important was the inclusion of the journal in PubMed Central, NLM’s digital journal repository [6], which also made all journal issues available in PubMed. PubMed coverage increased access to articles both via PubMedCentral and the journal site.

**Figure 2:**
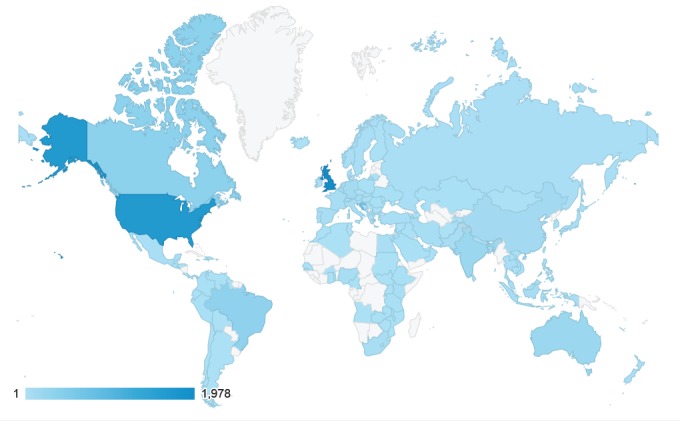
Journal's global impact: in the 18 months between December 2011 and May 2012, the website of *Journal of Global Health* was visited 6805 times, attracting visitors from 1233 cities in 128 countries (Source: Google Analytics©). The largest number of visits (n = 1978) was from United Kingdom, closely followed by the USA (n = 1669).

According to Google Analytics© breakdown of *JoGH* web–site traffic 12 months before (1 December 2011 to 30 November 2012) and 6 months after PubMed coverage (1 December 2012 to 31 May 2013), direct visits to journal's website (http://jogh.org) increased from about 10 to 17 visitors per day, with a substantial growth in the category of “new visitors” (58.7% vs 71.2% before and after PubMed coverage, respectively). Daily maximum increased from 35 to 48 visits, and daily minimum from 1 to 4 visits. PubMed coverage also facilitated a truly global access to the journal's website. In total (18 months between Dec 2011 and May 2012, the website has been visited 6805 times, attracting visitors from 1233 cities in 128 countries (Source: Google Analytics©). The largest number of visits (n = 1978) was from United Kingdom, closely followed by the USA (n = 1669) (**Figure 2**).

**Figure 3:**
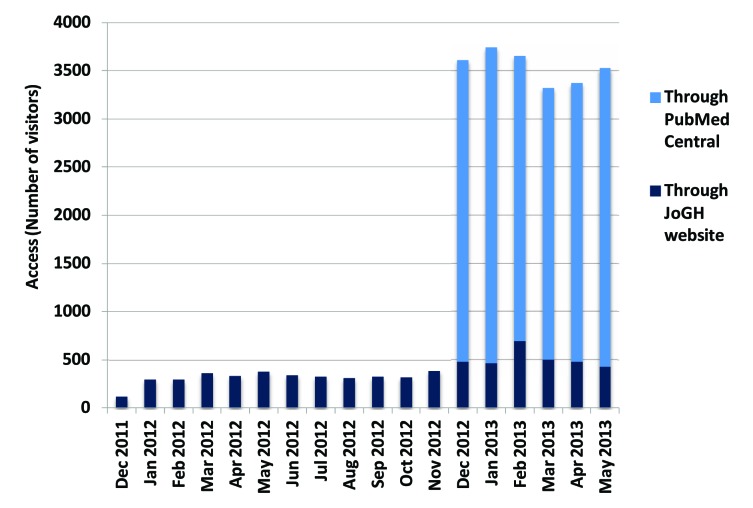
Access to the content of *Journal of Global Health* (*JoGH*), measured in number of visitors to the website (http://jogh.org) and number of visitors to journal’s PubMed Central site (December 2011 – May 2013).

The usage through PubMed directly is even more impressive (**Figure 3**). In the month when *JoGH* became available in PubMed Central (December 2012), the total number of visits to the journal’s content increased 10–fold, to more than 3500 visits per month in the first 3 months. The visits came from all over the world, and PubMed coverage increased those from developing countries. A total of 18 194 requests for full–text articles were recorded through PubMed Central within the first six months (December 2012 – May 2013).

**Figure:**
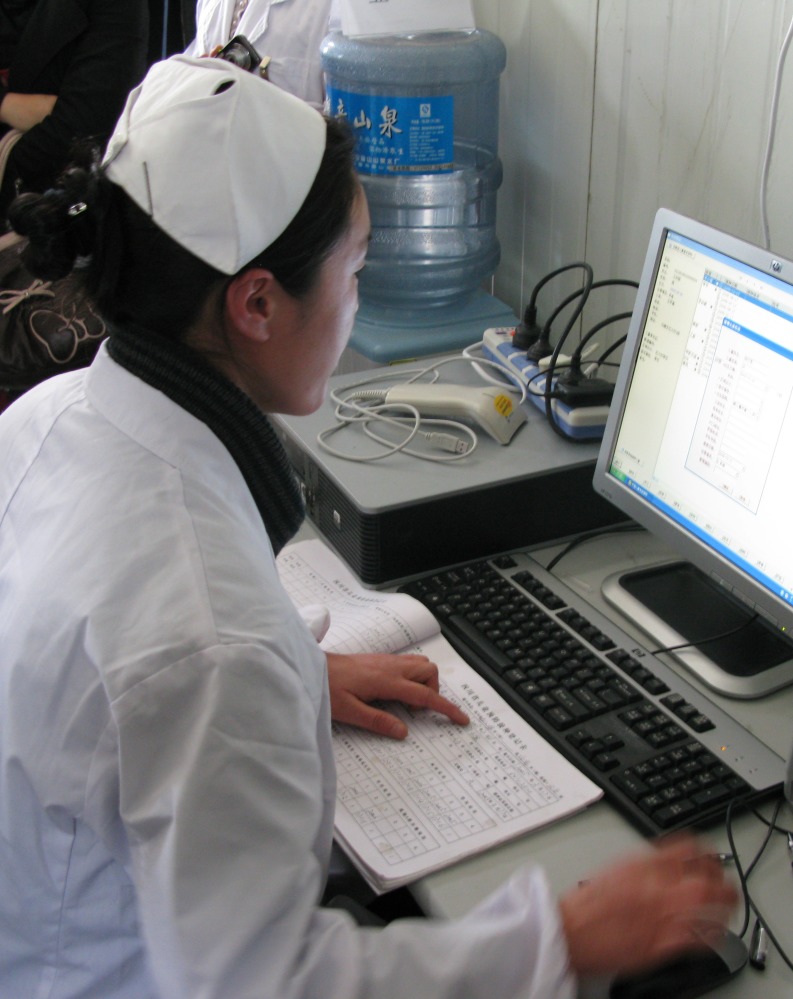
Photo: Courtesy of David Hipgrave, personal collection

The top 10 most accessed and downloaded articles during the first six months (December 2012 – May2013) of *JoGH*'s availability via PubMed are shown in **Table 1**. The authors of these papers come from different countries, both high–income and low– and middle–income countries, and different world regions.

**Table 1:** Top 10 most accessed articles in *Journal of Global Health* via PubMed Central, December 2012 – May 2013

Rank	Author	Title	Full text	PDF download	Total requests
1	Meem M et al.	Biomarkers for diagnosis of neonatal infections: A systematic analysis of their potential as a point–of–care diagnostics	1068	677	1745
2	Jawad I et al.	Assessing available information on the burden of sepsis: global estimates of incidence, prevalence and mortality	749	352	1101
3	Buckle GC et al.	Typhoid fever and paratyphoid fever: Systematic review to estimate global morbidity and mortality for 2010	807	283	1090
4	Roberts T et al.	Epidemiology and aetiology of maternal parasitic infections in low– and middle–income countries	635	364	999
5	Bahl R et al.	Setting research priorities to reduce global mortality from preterm birth and low birth weight by 2015	619	315	934
6	Waters D et al.	Aetiology of community–acquired neonatal sepsis in low and middle income countries	607	231	838
7	PalaniVelu P et al.	Epidemiology and aetiology of maternal bacterial and viral infections in low– and middle–income countries	589	174	763
8	Torti J	Floods in Southeast Asia: A health priority	654	61	715
9	Hipgrave D	Communicable disease control in China: From Mao to now	526	146	672
10	Kolčić I	Double burden of malnutrition: A silent driver of double burden of disease in low– and middle–income countries	429	111	540

The interest of *JoGH* readers is clear – they want high–quality systematic reviews and viewpoints on those issues that are relevant for the global community, followed by regional issues with a global relevance and impact (**Table 1**). In future, *JoGH* will continue its mission of publishing high–quality peer–reviewed original research, objective reviews and personal viewpoints of global health and development issues [7]. As a member of the Committee of Publication Ethics (COPE), *JoGH* will keep follow the best practices in scientific publishing and ensure the integrity of the published record [8].

## PROPOSAL TO MEDLINE/PubMed

The analysis of records in different databases of the largest global medical library, NLM, clearly demonstrated how a phenomenal increase in global health research has not been followed by systematic conceptualization in recording the publication output from this research, as well as capturing sources of global health research information. I propose the inclusion of “global health” as a MeSH term – to recognize the new research discipline in health. This will greatly assist in finding relevant evidence about global health issues and facilitate the translation of knowledge to practice at all levels of health care.

## References

[1] Koplan JP, Bond T, Merson M, Reddy K, Rodriguez M, Sewankambo N (2009). Towards a common definition of global health.. Lancet.

[2] Niblett DH (1966). Global health factors of importance to Canadian mobile forces with a potential world-wide commitment.. Med Serv J Can.

[3] von Bahr G (1976). Blindness prevention – a global health problem. Lakartidningen.

[4] Panosian C, Coates T (2006). The new medical “missionaries”: grooming the next generation of global health workers.. N Engl J Med.

[5] US National Library of Medicine. Fact Sheet. Medical Subject Headings (MeSH®) Available at: http://www.nlm.nih.gov/pubs/factsheets/mesh.html Accessed: 29 May 2013.

[6] US National Library of Medicine. Fact Sheet. MEDLINE, PubMed, and PMC (PubMed Central): How are they different? Available at: http://www.nlm.nih.gov/pubs/factsheets/dif_med_pub.html Accessed: 29 May 2013.

[7] (2011). Journal of Global Health. Mission statement.. J Glob Health.

[8] Wager E (2012). The Committee on Publication Ethics (COPE): Objectives and achievements 1997-2012.. Presse Med.

